# Durable Response to Redifferentiation in a Patient With Metastatic *NCOA4::RET* Fusion-driven Papillary Thyroid Cancer

**DOI:** 10.1210/jcemcr/luaf054

**Published:** 2025-05-05

**Authors:** Valentina D Tarasova, Christine H Chung, Sarimar Agosto Salgado

**Affiliations:** Department of Head and Neck-Endocrine Oncology, Moffitt Cancer Center, Tampa, FL 33612, USA; Department of Head and Neck-Endocrine Oncology, Moffitt Cancer Center, Tampa, FL 33612, USA; Department of Head and Neck-Endocrine Oncology, Moffitt Cancer Center, Tampa, FL 33612, USA

**Keywords:** selpercatinib, *RET* fusion, redifferentiation, RAI-refractory thyroid cancer

## Abstract

Redifferentiation therapy (RDT) is a promising strategy for follicular cell-derived thyroid cancer (TC) in the era of personalized oncology. Limited data are available on long-term clinical outcomes of RDT in patients with fusion-driven TC. A 22-year-old female with recurrent radioactive iodine (RAI)-refractory metastatic progressive, *NCOA4::RET* fusion-driven papillary TC was treated with selective *RET* inhibitor, selpercatinib, for 3 months before a therapeutic dose of RAI 146 mCi (5402 MBq). The posttherapy scan showed enhancement of RAI avidity of previously mildly avid pulmonary metastases. After RDT, the thyroglobulin levels significantly declined, and pulmonary nodules completely resolved on chest computed tomography scan. At 24 months of follow-up, the patient did not have evidence of progression. Moreover, thyroglobulin levels continued to decline. RDT with selpercatinib enhanced RAI uptake in the lungs, with a persistent structural and biochemical response sustained 24 months after RAI therapy and discontinuation of selpercatinib. Clinical trials are warranted further to investigate RDT with selective inhibitors in oncogene fusion-driven TC.

## Introduction

MAPK pathway activation is a cornerstone of follicular cell-derived thyroid cancer (TC) development and progression that leads to inhibition of sodium iodine transporter and loss of the ability of TC cells to concentrate iodine [1]. Redifferentiation therapy (RDT) is based on restoring radioactive iodine (RAI) uptake after a short course of pharmacological inhibition of the MAPK pathway with consecutive treatment with RAI [1, 2]. The RDT concept was tested in preclinical studies and later in a proof-of-principle clinical trial of mitogen-activated extracellular signal regulated kinase (MEK) inhibitor [3, 4]. Response to RDT differed significantly according to the driver pathogenic variant [4-7]. RDT with *BRAF V600E* inhibitors with or without MEK inhibitors restored RAI uptake in 60% to 95% of patients with a 6-month radiological response of up to 38% [5, 8, 9]. *RAS*-mutated TC had restored RAI uptake after RDT in 66% to 100% with a 6-month radiological response in 20% [4-7].

Fusion-driven TC represents less than 10% of all TC. Selpercatinib is the only targeted therapy approved for *RET* fusion-driven TCs; other targeted therapies have been approved for *NTRK* and *ALK* gene fusion-positive solid tumors. Only a few patients treated with RDT in fusion-driven TC are reported. RDT with *NTRK* inhibitor larotrectinib was reported in 4 patients with *NTRK* fusion TC. Three of 4 patients restored uptake on RAI diagnostic whole-body scan (WBS) [10-12]. One patient with *ALK* fusion-driven TC did not show restoration of RAI uptake after RDT with alectinib [6]. However, RDT of *ALK* fusion-driven TC showed promising outcomes in preclinical studies [13].

To date, RDT in patients with *RET* fusion-driven TC has been reported in 12 patients (including 2 pediatric patients). Four patients demonstrated an increase in RAI uptake in distant metastases after the use of selective *RET* inhibitors, selpercatinib or pralsetinib [6, 14-18]. Two of 3 patients with *RET* fusion-driven TC restored RAI uptake after selumetinib therapy [4]. One of 3 patients with *RET* fusions in the Mayo cohort restored RAI uptake, and 2 did not redifferentiate with selpercatinib and pralsetinib [6]. Specific fusions were reported in 3 of 12 patients: *NCOA4::RET* and 2 *CCDC6::RET*, and all showed response to RDT [12, 14, 15]. Therefore, the published data have provided evidence of restoration in the I-131 uptake in the metastatic sites with redifferentiation protocols. However, long-term outcomes of RDT in *RET* fusion-driven papillary thyroid carcinoma (PTC) have not been described.

To contribute to the limited knowledge of redifferentiation in *RET* fusion-driven TC, we present a case of a woman with *NCOA4::RET* fusion RAI refractory (RAIR) PTC who underwent treatment with a selective *RET* inhibitor, selpercatinib. This treatment enhanced RAI uptake in lung metastases with a sustained response 24 months after RAI therapy and discontinuation of selpercatinib.

## Case Presentation

A 22-year-old woman presented with a progressive RAIR PTC, with a local neck recurrence and metastases to the lungs. She initially felt a lump in her neck at age 19 years. A fine-needle aspiration biopsy of the isthmus nodule showed Bethesda category VI, PTC. There was no known family history of TC.

Preoperative evaluation revealed bilateral bulky lymphadenopathy on neck ultrasound and computed tomography (CT) neck. The patient underwent total thyroidectomy with central and bilateral lateral neck dissections. Pathology demonstrated multifocal PTC, classic and solid subtypes, the largest focus measuring 2.8 cm. Lymphatic invasion identified no vascular invasion. The surgical margins were negative. Forty-five of 90 lymph nodes (LN) were positive for PTC, with the largest metastatic focus at 4.5 cm with extranodal extension. TC was at high risk for recurrence based on the American Thyroid Association risk stratification [19]. At the time of diagnosis, the cancer was stage I, pT2, N1b, M0 (AJCC 8th edition), and there was no clear evidence of lung metastasis assessed by CT scan of the chest.

**Figure 1.**
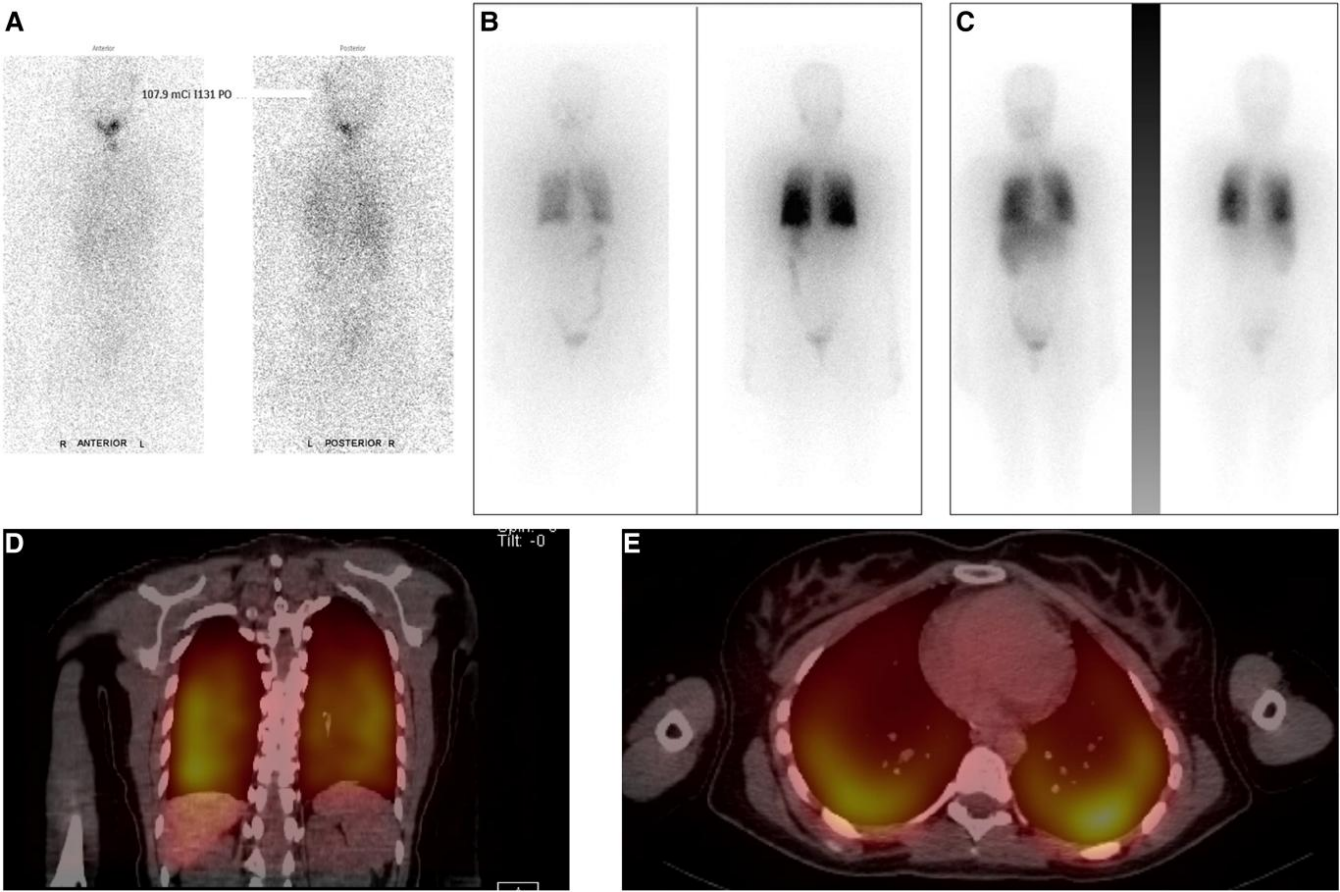
Imaging describing the response to radioactive iodine therapy at baseline and after redifferentiation with selpercatinib. (A) I-131 posttherapy whole-body scan (WBS) after the first dose of radioactive iodine (RAI) (I-131 107.9 mCi [3992.3 MBq]) shows uptake in the neck and mild diffuse uptake in the lungs on the posterior images. No evidence of RAI-avid skeletal metastasis. (B) Pretherapy WBS I-123 5.9 mCi (218 MBq) shows no focal residual radioiodine uptake in the neck/thyroid bed. There is moderate to intense radioactive iodine accumulation outlining the entire lung parenchyma and best appreciated on posterior view, appearing as the most intense site of uptake and with a significant fraction of the total visible dose distribution. (C) Posttherapy WBS I-131 146 mCi (5402 MBq) shows similar to pretreatment scan intense diffuse uptake throughout both lungs. The patient has minimal uptake in the neck/thyroid bed. The patient was given 14 mCi (518 MBq) I-131 orally before uptake. The 21-hour radioactive iodine uptake is 4%. (D, E) Single-photon emission computed tomography (SPECT-CT) on posttreatment scan showed mild/intense diffuse uptake in the lungs.

Two months after the initial surgery, she was treated with RAI 107.9 mCi (3992.3 MBq) with recombinant human TSH (rhTSH). She followed the low-iodine diet for 3 weeks before therapy. Stimulated thyroglobulin (TG) levels were not available. Posttreatment WBS showed uptake in the thyroid bed and mild diffuse uptake in the lungs on the posterior images. There was no evidence of RAI-avid skeletal metastasis (Fig. 1A).

Three months after RAI therapy, surveillance revealed recurrent/persistent disease in the left neck. TG level increased to 659 ng/mL (659 µg/L) (normal range: 2.8-40.9 ng/mL; 2.8-40.9 µg/L) with negative TG antibodies (AB) and TSH of 0.78 µIU/mL (0.8 mIU/L) (normal range: 0.39-4.94 uIU/mL; 0.4-4.9 mIU/L). The patient underwent revision surgery-left lateral neck dissection. Pathology demonstrated 10 of 24 resected LN with metastatic PTC; the largest metastatic deposit was 1.5 cm without extranodal extension. TG levels decreased to 390 ng/mL (390 µg/L) with TSH 0.99 µIU/mL (1 mIU/L) after the second surgery, and TG AB remained undetectable. The patient was referred to a tertiary academic center.

## Diagnostic Assessment

Next-generation sequencing of the resected tumor detected *NCOA4::RET* fusion. Germline genetic testing showed a variant of uncertain significance in the cell-cycle checkpoint kinase 2 (*CHEK2*) gene.

**Figure 2.**
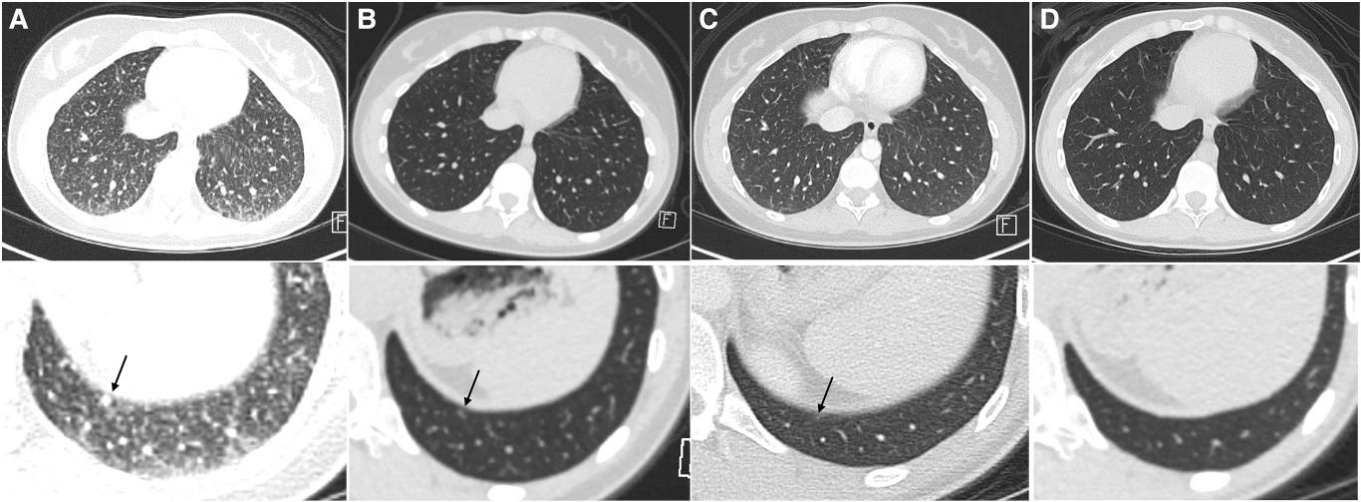
Computed tomography (CT) scan of the chest demonstrating changes in lung metastases after selpercatinib and radioactive iodine therapy. (A) CT scan of the chest with contrast before selpercatinib shows innumerable lung metastases (miliary pattern), with the dominant pulmonary nodule measuring 0.6 cm. The arrow points to one of the dominant subpleural solid nodules (0.6 cm) in the left lower lobe. (B) CT scan of the chest without contrast after 3 months of selpercatinib showed improvement/resolution of punctate pulmonary nodularity seen on the prior examination, consistent with favorable interval response to therapy. The arrow indicates a 3-mm nodule in the left lower lung. (C) CT scan of the chest with contrast 12 months after redifferentiation with selpercatinib and RAI 146 mCi (5402 MBq) shows a stable 3-mm subpleural pulmonary nodule. The arrow points to the 3-mm nodule in the left lower lung. (D) CT scan of the chest with contrast 24 months shows resolution of miliary pulmonary metastases.

Follow-up surveillance imaging with a CT scan of the neck with contrast revealed bilateral suspicious subcentimeter LN, including retropharyngeal, diagnosed just 2 months after revision surgery. Furthermore, CT scan of the chest showed diffuse numerous miliary pulmonary nodules measuring up to 0.6 cm, consistent with metastatic disease (Fig. 2A). Comprehensive imaging revealed no additional metastatic sites. The patient denied any respiratory symptoms from pulmonary metastases, and there was no clinical evidence of hypoxemia. Her disease was restaged at stage II, pT2, N1b, M1 (AJCC 8th edition).

## Treatment

Multidisciplinary consensus recommended RDT with selpercatinib considering the rate of progression and clinical concerns for RAIR TC. The patient was treated with selpercatinib 160 mg twice per day for 3 months. The patient tolerated selpercatinib without declining performance or developing any grade 2 or 3 adverse events. The patient had grade 1 nausea and loose stools. TG level decreased from 357 ng/mL (357 µg/L) to 82.1 ng/mL (82.1 µg/L) and 79.8 ng/mL (79.8 µg/L) while on selpercatinib therapy (Table 1). Chest CT showed improvement and resolution of punctate nodularity seen on the prior scan after 3 months of selpercatinib (Fig. 2B).

**Table 1.** Thyroglobulin changes over time before, during, and after redifferentiation

Laboratory test	Reference range	Time after redifferentiation (mo)									
		−3	Selpercatinib			RAI/rhTSH	5	9	12	18	24
			1	2	3						
TSH	0.39-4.94 µIU/mL (0.39-4.94 mIU/L)	0.99 µIU/mL (0.99 mIU/L)	0.3 µIU/mL (0.3 mIU/L)	1.06 µIU/mL (1.06 mIU/L)	0.43 µIU/mL (0.43 mIU/L)	83.3 µIU/mL (83.3 mIU/L)	0.08 µIU/mL (0.08 mIU/L)	0.11 µIU/mL (0.11 mIU/L)	0.05 µIU/mL (0.05 mIU/L)	0.08 µIU/mL (0.08 mIU/L)	0.33 µIU/mL (0.33 mIU/L)
TG*^a^*	2.8-40.9 ng/mL (2.8-40.9 µg/L)	390 ng/mL (390 µg/L)	357 ng/mL (357 µg/L)	81.1 ng/mL (81.1 µg/L)	79.8 ng/mL (79.8 µg/L)	174 ng/mL (174 µg/L)	22.4 ng/mL (22.4 µg/L)	20 ng/mL (20 µg/L)	19.3 ng/mL (19.3 µg/L)	17.3 ng/mL (17.3 µg/L)	14.7 ng/mL (14.7 µg/L)

Abbreviations: RAI, radioactive iodine; rhTSH, recombinant human TSH; TG, thyroglobulin.

^a^Thyroglobulin antibodies remained negative through the clinical course.

The patient was treated with the second dose of I-131 146 mCi (5402 MBq) with rhTSH. The pretreatment scan showed a diffuse moderate to intense uptake throughout both lungs (Fig. 1B). Post-RAI therapy WBS showed a pattern and distribution of I-131 similar to the pretreatment scan (Fig. 1C-1E). After RAI therapy, she had grade 1 dysgeusia and nausea. The patient discontinued selpercatinib 2 days after the RAI treatment dose. TG stimulated to 174 ng/mL (174 µg/L) with a TSH of 88.3 µIU/mL (83 mIU/L) after rhTSH (Table 1).

## Outcome and Follow-Up

The patient had a sustained favorable biochemical response to RAI with TG decreasing to 22.4 ng/mL (22.4 µg/L), 20 ng/mL (20 µg/L), 19.3 ng/mL (19.3 µg/L), 17.3 ng/mL (17.3 µg/L), and 14.7 ng/mL (14.7 µg/L) at 5, 9, 12, 18, and 24 months after RDT with suppressed TSH (Table 1). TG AB remained undetectable. Chest CT showed a decrease in the size of the dominant lung nodule to 3 mm at the 12-month follow-up and a complete resolution of pulmonary nodules at the 18-month follow-up (Fig. 2C). Complete radiological resolution of punctate nodularity in the lungs and neck was maintained at 24 months (Fig. 2D). There were no suspicious neck LNs on the CT scan of the neck from 5 to 24 months after RDT. Overall, the patient reported no sequelae from therapy and remained asymptomatic 24 months after the last dose of RAI and discontinuation of selpercatinib.

## Discussion

RDT with selective *RET* inhibitors is a promising treatment strategy for patients with *RET* fusion-driven PTC. Here, we described a course of an aggressive *NCOA4::RET*-driven RAIR PTC in a woman in whom lung tumors improved the ability to concentrate RAI, including subsequent durable response to RDT therapy for 24 months. This is the first case report of a sustained response of 24 months to RDT after discontinuing selpercatinib in an *NCOA4::RET*-driven RAIR PTC. We hypothesize that clinical outcomes were achieved by combining the antineoplastic effects of selpercatinib and the cytotoxic effects of I-131 on TC cells. There is the possibility the results are secondary to a durable radiological response after a 3-month selpercatinib therapy. However, the change in uptake in the lungs from mild to moderate and intense and elevated TG after selpercatinib supports redifferentiation in the lungs, and, therefore, the durable response can result from a combination of targeted therapy and RAI. Prospective clinical trials of selective *RET* inhibitors in *RET* fusion-driven PTCs are needed to address these questions (NCT05668962, NCT06458036).

As previously reported, cases show heterogeneous responses to RDT in *RET* fusion-driven TC, it is important to identify markers of response and resistance to RDT. SWI/SNF gene mutation may be a potential marker of resistance to RDT [20]. Furthermore, the response according to the patient's age and specific *RET* fusions warrants further exploration in clinical trials.

The optimal duration of RDT remains uncertain. Most RDT clinical trials used the redifferentiation agents for 3 to 6 weeks. There may be a potential benefit to extending the duration of RDT, if well tolerated, to maximize the effect of RAI. We hypothesize that a small disease burden in our patient may have resulted in better clinical outcomes.

The effect of RDT on the specific location of metastasis is unknown. RDT may have different efficacy rates among different disease sites. RDT as an adjuvant therapy was investigated in ASTRA clinical trial in patients at high risk of recurrence [21]. The study did not meet the primary endpoint with statistical significance; however, the concept of RDT in the adjuvant setting can be rechallenged, particularly in patients with distant metastasis who are RAI-naïve but unlikely to respond to RAI. Also, a combination of RDT and dual inhibition of the MAPK pathway may be more effective [20].

There is a concern that RDT may lead to the selection of aggressive clones that may lead to inferior long-term clinical outcomes [6]. Prospective studies and longer follow-ups are warranted to assess RDT's safety and potential for a cure.

Management of TC becomes individualized and tumor-agnostic because of the advancements in understanding TC genomics, the development of various targeted systemic therapies, and the availability of quality data from prospective clinical trials. In addition, the selection of candidates for RAI therapy has evolved as clinicians recognize the effects of TC genomics on sensitivity to RAI as well as the longitudinal side effects of RAI. RDT may be a valuable contributor to treating patients with follicular cell-derived TC; however, clinical studies are warranted to determine the ideal target patient population, time of initiation, selection, and duration of RDT agents, markers of response and resistance, and frequency of RDT.

## Learning Points

- Selective *RET* inhibitor-selpercatinib can be used for redifferentiation in patients with *RET* fusion-driven RAIR TC.
- Enhancement of RAI avidity in the lungs on posttherapy scan correlated with long-term response to RDT.
- An incomplete biochemical response 24 months following RDT does not indicate a cure, warranting ongoing follow-up and investigation in clinical trials.
- Efficacy and safety of RDT with selective inhibitors in fusion-driven cancers (*RET, ALK, NTRK*) should be further investigated in prospective clinical trials in salvage and adjuvant settings.

## Contributors

All authors made individual contributions to authorship. V.T. conceptualized the idea of the case report and prepared the manuscript draft. V.T., S.A.S., and C.H.C. prepared and edited the manuscript. V.T. and S.A.S. were involved in the diagnosis and management of this patient. All authors reviewed and approved the final draft.

## Data Availability

Data sharing is not applicable to this article as no datasets were generated or analyzed during the current study.
